# P-971. Adaptation of an Antimicrobial Scoring System to Optimize Prospective Audit and Feedback in a Large Academic Pediatric Hospital

**DOI:** 10.1093/ofid/ofaf695.1170

**Published:** 2026-01-11

**Authors:** Karisma Patel, Brandon Monroe, Sarah E Firmani, Khyati Amin, Mehgan Kidd

**Affiliations:** Children's Health, Dallas, TX; Dallas Childrens, mesquite, Texas; Children's Health, Dallas, TX; Children's Medical Center - Dallas, Dallas, TX; University of Texas Southwestern Medical Center Dallas, Dallas, Texas

## Abstract

**Background:**

Prospective audit and feedback (PAF) is a nationally recognized core strategy of antimicrobial stewardship (ASP) programs. Given the growing responsibilities of ASP programs and high patient volumes, methods to improve the efficiency of the PAF process using information technology are needed.

**Table 1.**
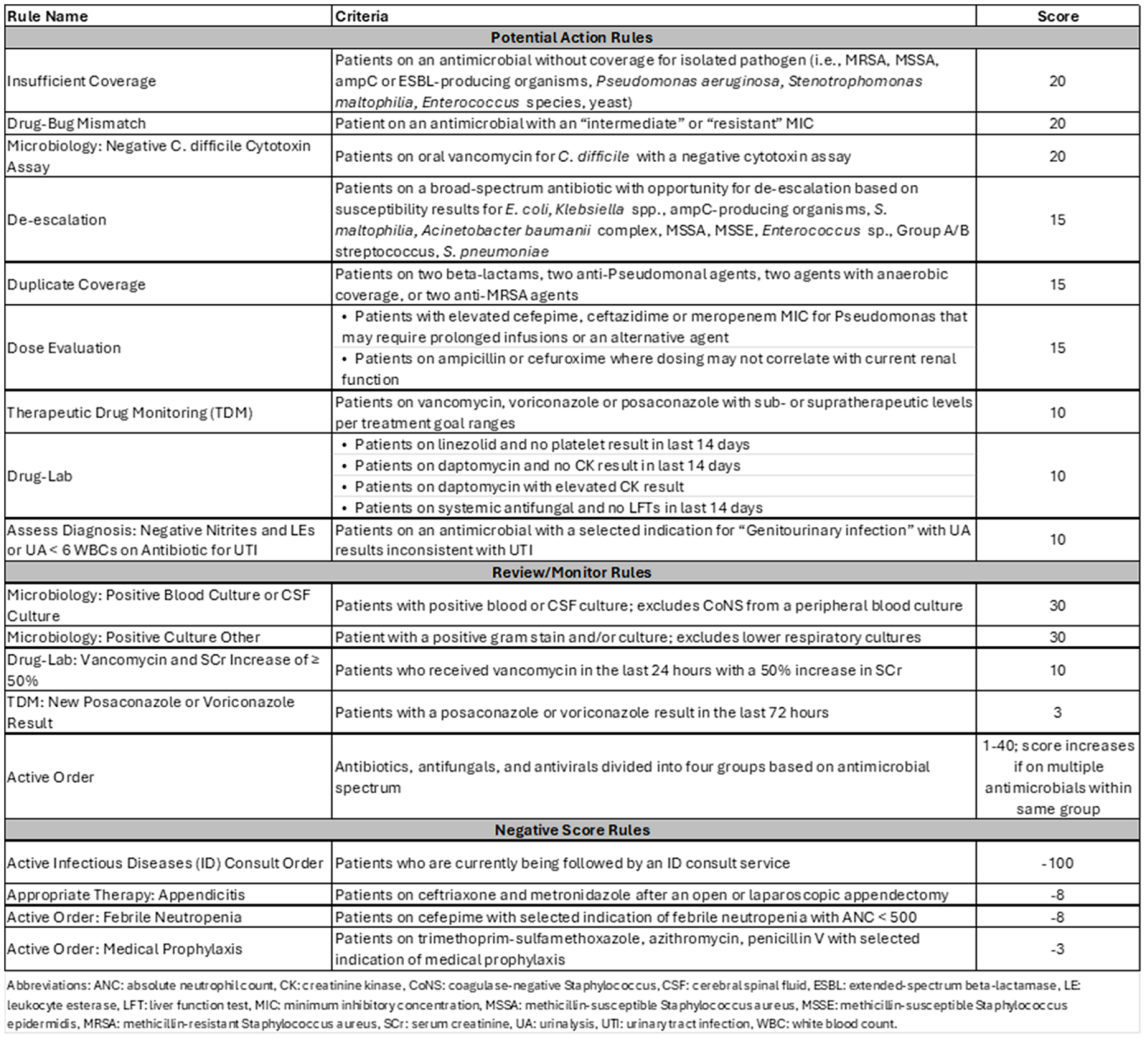
Antimicrobial Stewardship Scoring System Rules

**Table 2.**
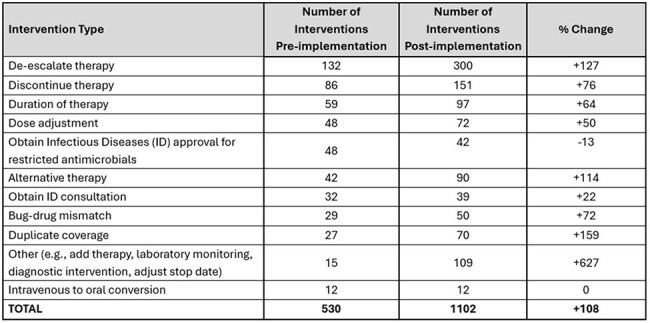
Frequency of Prospective Audit and Feedback Intervention Types

**Methods:**

The ASP program at Children’s Medical Center Dallas began conducting PAF of patients on any antimicrobial active for at least 48-72 hours in 2018. Patients were identified using a manual report in Epic and listed indiscriminately. In July 2022, “handshake stewardship” was initiated in the intensive care units (ICU), where all patients on antimicrobials were reviewed daily regardless of antimicrobial duration. In April 2023, a scoring system in Epic was implemented to identify patients for PAF. This tool used real-time objective data to assign scores based on intervention potential and acuity, irrespective of antimicrobial duration. Foundational rules were customized, and new rules were created to account for pediatric considerations (Table 1). Multiple rules could trigger simultaneously to create a composite score (Figure 1). Patients were listed in descending order based on score. Once addressed, any rule could be deferred for a set time to allow for score reduction. We compared the number and types of interventions pre- and post-implementation of the scoring system from January-December 2019 to October 2023-September 2024. Two ASP team members performed daily reviews in each period.

**Figure 1.**
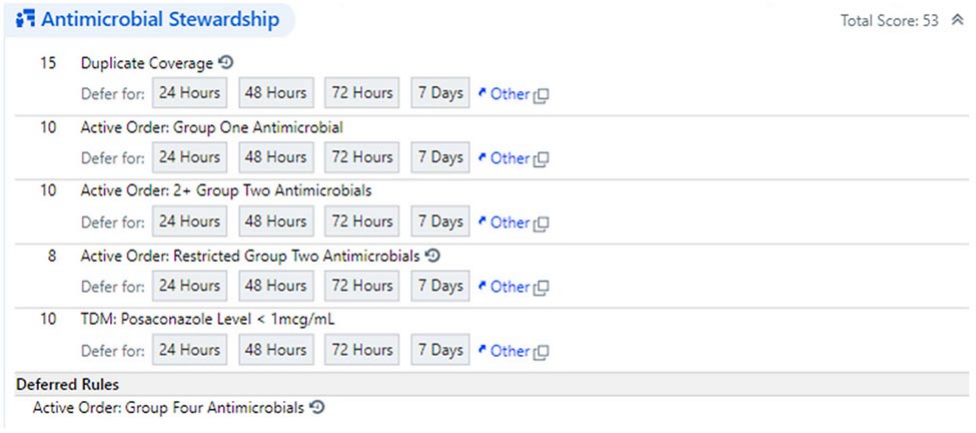
Example of Epic Antimicrobial Stewardship Scoring and Rules

**Figure 2.**
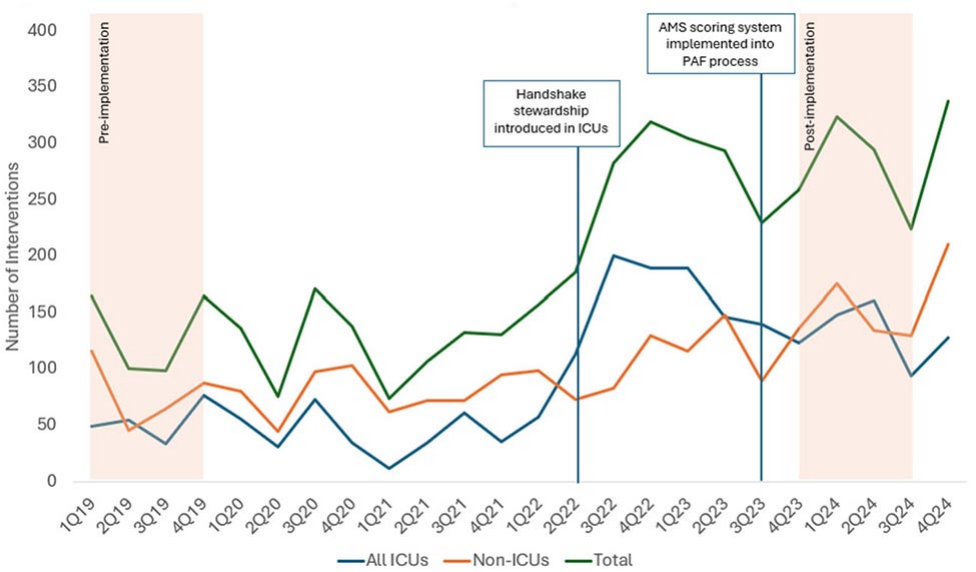
Trend in Number of Prospective Audit and Feedback Interventions (2019-2024)

**Results:**

A total of 530 interventions were made from 8,503 patient reviews (6% intervention rate) in the pre-implementation period versus 1,102 interventions from 6,916 patient reviews (16% intervention rate) post-implementation. The number of interventions for ICU and non-ICU services increased from 215 to 526 (145% increase) and 315 to 576 (83% increase), respectively (Figure 2). The most common interventions were to de-escalate therapy, discontinue therapy or adjust the duration of therapy in both periods (Table 2).

**Conclusion:**

Customizing Epic’s integrated antimicrobial scoring system can increase efficiency and prioritize high-impact interventions of ASP teams performing daily PAF in pediatric patients.

**Disclosures:**

All Authors: No reported disclosures

